# 

**Published:** 1840-07

**Authors:** 


					﻿THE
WESTERN JOURNAL
OF
MEDICINE AND SURGERY.
COLLABORATORS.
Edward H. Barton, M. D. Profes
sor of the Theory and Practice o,
Medicine, in the Medical College o,
Louisiana.
William J. Barbee, M. D. George
town, Ky.
George W. Bayless, M. D. Dissector
for the Pathological Cabinet of th
Louisville Medical Institute.
A- H. Buchanan, M. D. Columbia
Tennessee.
Charles Caldwell, M. D. Professoi
of the Institutes of Medicine anc
Medical Jurisprudence, in the Lou
isville Medical Institute.
Samuel A. Cartwright, M. D. Mis-
sissippi.
A. Clapp, M. D. New Albany, In-
diana.
Jedediah Cobb, M. D. Professor 0/
Anatomy, in the Louisville Medical
Institute.
John Esten Cooke, M. D. Professor
of the Theory and Practice of Medi-
cine, in the Louisville Medical In-
stitute.
Samuel D. Gross, M. D. Professor of
Surgery in the Louisville Medical
Institute.
John Hardin, M. D. Munfordsville,
Ky.
John P. Harrison, M. D. late Profes-
sor of Materia Medica, in the Cin-
cinnati College.
John F. Henry, M. D. Bloomington,
Illinois.
Samuel P. Hildreth, M. D. Marietta,
Ohio.
Samuel Hogg, M. D. Nashville, Ten-
nessee.
M. Z. Kreider, M. D., Lancaster,
Ohio.
Moses L. Linton, M. D. Springfield,
Kentucky.
Henry Miller, M. D. Professor of
Obstetrics and the Diseases of Wo-
men and Children, in the Louisville
Medical Institute.
John W. Monette, M. D. Washing-
ton, Mississsippi.
Henry Perrine, M. D. Florida.
Samuel B. Richardson, M. D. Lou-
isville, Ky.
John L. Riddell, M. D. Professor of
Chemistry in the Medical College
of Louisiana.
Landon C. Rives, M. D. late Pro-
fessor of Obstetrics, in the Medical
Department of the Cincinnati Col-
lege.
Charles W. Short, M. D. Professor
of Materia Medica, in the Louisville
Medical Institute.
G. Troost, M. D. Professor of Chem-
istry and Mineralogy, in the Uni-
versity of Nashville.
Amasa L. Trowbridge, M. D. Pro-
fessor of Surgery, Willoughby Uni-
versity, Ohio.
John A. Warder, M. D. Cincinnati,
Ohio.
William Wood, M. D. Cincinnati,
Ohio.
The Western Journal oe Medicine and Surgery is published monthly by
the undersigned, at the corner of Main and Fifth streets, Louisville, at $5 per
annurn, payable in advance. Each number contains from 80 to 84 pages making
two volume^ in the year of about 500 pages.
Contributions'to its pages by the Physicians of the Valley of the Mississippi,
addressed to the Editors, are respectfully'solicited.
Letters on business, to be addressed, postage paid, to the publishers.
July 25, J840.	‘	' PRENTICE & WEISSINGER.
				

